# Molecular Basis of Rare Inherited Tubulopathies of the Kidney: A Primer for Clinicians

**DOI:** 10.3390/ijms27093940

**Published:** 2026-04-28

**Authors:** Marta Vecino-Pérez, María García-Murias, Noa Carrera, Pablo Pedrosa, Miguel Á. García-González

**Affiliations:** 1 Group of Genetics and Developmental Biology of Renal Disease, Laboratory of Nephrology, Health Research Institute of Santiago de Compostela (IDIS), Clinical University Hospital of Santiago de Compostela (CHUS), 15706 Santiago de Compostela, Spain; marta.vecino.perez@sergas.es (M.V.-P.); maria.garcia.murias@sergas.es (M.G.-M.); miguel.garcia.gonzalez@sergas.es (M.Á.G.-G.); 2 RICORS2040 (Kidney Disease), Instituto de Salud Carlos III (ISCIII), 15706 Santiago de Compostela, Spain

**Keywords:** hereditary tubulopathies, rare renal tubulopathies, monogenic kidney diseases, electrolyte transport disorders, dent disease, Bartter syndrome, Gitelman syndrome, Liddle syndrome

## Abstract

Hereditary renal tubulopathies are rare monogenic disorders caused by defects in tubular transport mechanisms that impair the handling of electrolytes, water, and acid–base balance along the nephron. While each tubulopathy is individually uncommon, their collective burden is clinically relevant, as these disorders can severely affect quality of life and predispose to nephrolithiasis, dehydration episodes, and progression to chronic kidney disease. Advances in molecular genetics have identified more than 70 genes involved in renal tubular physiology; however, a substantial proportion of these cases remain genetically unresolved, and marked phenotypic heterogeneity complicates diagnosis and management. This narrative review provides an integrated overview of the main transport systems operating in the different tubular segments of the nephron—proximal tubule, thick ascending limb of the loop of Henle, distal convoluted tubule and collecting duct—summarizing the tubulopathies associated with each segment and discussing in greater detail representative inherited disorders that illustrate the clinical consequences of their dysfunction. We highlight current diagnostic challenges and limitations of existing therapeutic strategies and discuss novel diagnostic approaches as well as emerging treatment options. Improved genetic diagnosis, validation of candidate biomarkers, and the development of novel therapeutic strategies will be essential to advance precision medicine and improve outcomes for patients with inherited renal tubulopathies.

## 1. Introduction

Hereditary renal tubulopathies are rare monogenic diseases characterized by a primary impairment of renal function resulting from disrupted tubular transport mechanisms, thereby altering the normal physiology of the nephron and the collecting system. Patients’ quality of life can be severely affected, as these diseases can lead to electrolyte disturbances, dehydration episodes, renal lithiasis, and progression to chronic kidney disease (CKD). More than 70 genes have been described in the pathogenesis of tubulopathies, affecting several electrolyte channels, transporters, and other regulatory proteins that operate along the nephron.

Renal function relies on two sequential and tightly coordinated processes: glomerular filtration and tubular handling of the filtrated fluid. While the glomerulus determines the initial composition of the ultrafiltrate, the tubular segments of the nephron are responsible for the selective reabsorption and secretion of the solutes, water, and acid-base equivalents, ultimately defining urine composition.

Each of its segments—proximal tubule, loop of Henle, distal convoluted tubule, and collecting duct—has its own specific set of molecular transport mechanisms that, when disrupted, give rise to characteristic clinical and biochemical phenotypes. Although many tubulopathies have an identified monogenic cause, others remain genetically uncharacterized, reflecting the complexity of the renal tubular physiology and the current limitations of the diagnostic tools. Therefore, understanding the main transport systems of each nephron segment and the diseases associated with their malfunction is key to achieving an accurate diagnosis, improving patient management, and developing novel therapeutic strategies.

In this narrative review, we focus on hereditary renal tubulopathies affecting the different tubular segments of the nephron, highlighting the physiological transport mechanism operating in each segment and discussing representative monogenic disorders that illustrate the clinical consequences of their dysfunction (Table 1).

## 2. Proximal Tubule

### 2.1. Physiology and Molecular Basis of Disease

The proximal tubule (PT) is the first segment of the renal tubule immediately downstream from the glomerulus. Its main function is the massive reabsorption of glomerular filtrate components, playing an essential role in acid-base and fluid-electrolyte homeostasis. The PT reabsorbs several filtered solutes, such as 60–70% of sodium and water, 80% of bicarbonate, and 99% of glucose, amino acids, and proteins, so even minimal defects in PT handling can lead to severe systemic alterations [1].

**Figure 1:**
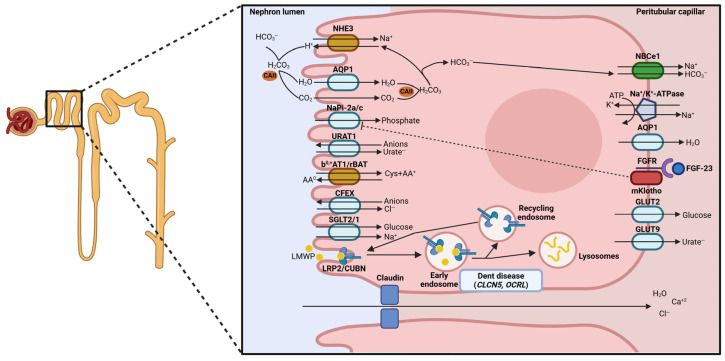
Main transport mechanisms from the proximal tubule epithelial cells, with the causal genes of Dent disease indicated as an example of tubulopathy affecting this nephron segment.

Along the different segments of the nephron, the movement of solutes relies primarily on active transport processes driven by the basolateral Na^+^/K^+^-ATPase, which maintains a low intracellular sodium concentration [2]. This activity creates an electrochemical gradient that drives the secondary active transport of multiple solutes through a wide range of cotransporters and exchangers, as well as paracellular pathways that allow the passive diffusion of ions and water following electrochemical gradients (Figure 1). Accordingly, Na^+^/K^+^-ATPase will be repeatedly discussed throughout the different sections of this review because of its essential role in renal electrolyte transport.

Sodium reabsorption from the PT lumen occurs mainly in exchange for protons via the Na^+^/H^+^ exchanger NHE3 (encoded by *SLC9A3*), and cotransport with organic solutes such as glucose and amino acids. Then, sodium experiences a secretion into the basolateral region of the tubule through the Na^+^/K^+^-ATPase [3].

Calcium is mainly reabsorbed in the PT (>60%), specifically through passive paracellular processes (90%) [2,3]. This calcium flow is due to the generation of a concentration gradient into the PT lumen, driven by the osmotic gradient created by water reabsorption through the movement of sodium [2]. The paracellular flow of calcium is allowed by the expression of claudins, specifically claudin-2, -10a, -12 and -17 (encoded by *CLDN2*, *CLDN10a*, *CLDN12*, and *CLDN17*), which are localized at the tight junctions of the PT [4].

The coordinated action of NHE3 and carbonic anhydrase II (encoded by *CA2*) allows the indirect reabsorption of filtered bicarbonate, a key process for acid-base homeostasis and systemic pH balance. The secreted protons from NHE3 action combine with bicarbonate (HCO_3_^−^), generating carbonic acid (H_2_CO_3_), which is catalyzed in the luminal space by carbonic anhydrase II, leading to the formation of CO_2_ and H_2_O. Reabsorbed water and freely diffused CO_2_ in the intracellular space of PT epithelial cells are catalyzed again by the carbonic anhydrase II [5]. The newly generated H_2_CO_3_ dissociates into protons and bicarbonate, which is reabsorbed into the peritubular space by the Na^+^/HCO_3_^−^ cotransporter NBCe1 (encoded by *SLC4A4*) [3]. The reabsorption of bicarbonate contributes to the generation of the gradient for the paracellular chloride reabsorption and as a consequence, generates an electrochemical gradient which favors the paracellular reabsorption of calcium as well [2]. When this process is altered by, for example, mutations in *SLC4A4* or *CA2*, the luminal pH of the proximal tubule is affected, leading to proximal renal tubular acidosis (OMIM #604278 and #2597309, respectively) [6,7].

Chloride reabsorption in the late PT is predominantly passive and follows the electrical gradient created by the Na^+^/K^+^-ATPase. In addition, transcellular movement of Cl^−^ also occurs via apical organic anion exchangers such as CFEX (encoded by *SLC26A6*) in exchange for oxalate, formate, sulfate, or bicarbonate [8].

Phosphate movement is also driven by the Na^+^/K^+^-ATPase-generated gradient and mediated by the cotransporters NaPi-2a (encoded by *SLC34A1*), responsible for 70-80% of the uptake, while the remaining depends on NaPi-2c (encoded by *SLC34A3*) [1,9]. The apical expression of these transporters is highly regulated by factors such as the parathyroid hormone (PTH), fibroblast growth factor 23 (FGF-23), the endopeptidase PHEX, and Klotho, which promote their trafficking between the plasmatic membrane and intracellular compartments to regulate phosphate uptake [9,10,11,12,13]. Mutations in *SLC34A1* have been identified to cause Fanconi renotubular syndrome 2, associated with hypophosphatemic rickets (OMIM #613388) [14]. Loss-of-function mutations in *SLC34A3* are responsible for hypophosphatemic rickets with hypercalciuria (OMIM #241530), together with increased serum concentration of 1,25-dihydroxyvitamin D [9]. These two clinical manifestations are key to differentiating this pathology from the autosomal dominant hypophosphatemic rickets (OMIM #193100), caused by mutations in FGF-23 [15] and the X-linked hypophosphatemic rickets (OMIM #307800), caused by mutations in PHEX [16].

Glucose is almost completely reabsorbed in the PT through the sodium-glucose cotransporters. SGLT2 (encoded by *SLC5A2*) is expressed in the early segments of the PT, where it completes almost 97% of the reabsorption of glucose [17,18]. In contrast, SGLT1 (encoded by *SLC5A1*), expressed in the late segment of PT, mediates the reabsorption of the residual glucose filtered from the earliest PT segments [17]. Therefore, less than 1% of filtered glucose leaves the PT, resulting in its absence in the final urine. So, alterations in the SGLT2 transporter led to familial renal glucosuria (OMIM #233100), characterized by the inability to reabsorb glucose from the urine, but with normal blood glucose levels [18,19].

Amino acids and other organic solutes are reabsorbed in the PT via specific cotransporters and antiporters. For example, B^0^AT1 transporter (encoded by *SLC6A19*) couples the uptake of sodium with the reabsorption of neutral amino acids (as glutamine) in the early portion of the proximal tubule. Impaired reabsorption of neutral amino acids due to mutations in *SLC6A19* causes Hartnup disorder (OMIM #234500), characterized by extrarenal manifestations such as ataxia, psychiatric disorders, and pellagra [20,21]. Acidic amino acids are reabsorbed by the EAAT3 cotransporter (encoded by *SLC1A1*), which transports aspartate and glutamate together with sodium ions and one proton [22]. However, other transporters do not couple the movement of amino acids with sodium. For example, the antiporter rBAT/B^0,+^AT1 (encoded by *SLC3A1* and *SLC7A9*), mediates the transport of cystine and dibasic amino acids (arginine, lysine, and ornithine) in exchange for neutral amino acids. Mutations affecting this heterodimeric transporter cause cystinuria (OMIM #220100), the most common inherited primary aminoaciduria characterized by the formation of cystine stones in the urinary tract [23,24,25].

The reabsorption of filtered low molecular weight (LMW) proteins in the PT is important to avoid the urinary loss of vitamin carrier proteins (vitamin D-binding protein (DBP), retinol-binding protein (RBP), hormones (PTH, insulin, angiotensin II), enzymes (lysozyme, cystatin C), lipoproteins (apolipoprotein B), immune-related proteins (α1-, β2-microglobulin, immunoglobulin light chains), and other proteins. This process depends on receptor-mediated endocytosis via the megalin (encoded by *LRP2*) and cubilin (encoded by *CUBN*) multiligand receptor complex, located in the brush border membrane and apical compartments of the early PT epithelial cells [26,27].

This endocytic process consists of the internalization of the receptor complex bonded with the protein ligand through invagination of the cell membrane, mainly forming clathrin-coated vesicles. Then, these structures fuse with the apical early endosomes where ligands are dissociated from the receptor complex thanks to the acidic pH of endosomes. Megalin, cubilin and other proteins from the complex are recycled back to the brush border membrane, whereas the internalized ligands are delivered to lysosomes for degradation [28]. Examples of tubulopathies caused by alterations in the endocytic receptor complex are the Donnai-Barrow syndrome (OMIM #222448), the Imerslund-Grasbeck syndrome (OMIM #261100 and #618882) and Dent disease (OMIM #300009 and #300555), caused by mutations in megalin, cubilin and its associated protein amnionless, the endosomal chloride channel ClC-5, and endolysosomal phosphatase OCRL, respectively [29,30,31,32].

The PT is also highly permeable to water, which moves following the osmotic gradient. Water reabsorption occurs mainly transcellularly (~70%) via aquaporin-1 (encoded by *AQP1*), which is expressed at the apical and basolateral membranes, while the remaining water flows paracellularly [33].

The PT also plays a role in kidney detoxification by regulating the levels of endogenous solutes, such as creatinine and uric acid, and exogenous compounds, such as drugs or organic acids and bases, through specific transport systems. This group is mainly composed of members of the *SLC22* transporter subfamily, which encode organic anion transporters (OATs) expressed in both the basolateral and apical membranes of PT epithelial cells. As an example, URAT1 (encoded by *SLC22A12*), expressed in the apical membrane, reabsorbs filtered urate from the renal lumen via the exchange with organic and inorganic anions. So, loss-of-function mutations have been identified to cause hereditary renal hypouricemia (OMIM #220150), characterized by uric acid wasting in urine leading to acute kidney injury or nephrolithiasis [34].

Therefore, genetic defects affecting this nephron segment result in PT dysfunction syndromes characterized by the urinary loss of multiple solutes and a broad spectrum of clinical manifestations. Within the group of proximal tubular dysfunction syndromes, Dent disease is discussed in detail as a paradigmatic example of genetically driven impairment of PT endocytosis.

### 2.2. Dent Disease

Dent disease is an X-linked disorder characterized by a PT dysfunction that leads to the development of LMW proteinuria, which is the main clinical manifestation [32]. Molecules such as α1-microglobulin, β2-microglobulin, RBP, DBP, cystatin C, or PTH experience urine wasting due to the lack of reabsorption by the tubular epithelial cells. The loss of these proteins is associated with additional symptoms, such as hypercalciuria, aminoaciduria, phosphaturia, glycosuria, uricosuria, kaliuresis, impaired urinary acidification, nephrocalcinosis, and nephrolithiasis [35]. Patients may also develop secondary bone manifestations, such as rickets or hypercalciuric osteomalacia, due to chronic tubular losses. Typical symptoms are generally found in boys under 10 years old, who experience progressive renal failure leading to end-stage kidney disease (ESKD) in 30-80% of the cases from the ages of 30 to 50 [36].

Additionally, this disorder can be classified into two distinct types based on the affected gene. The *CLCN5* gene is responsible for 60% of the cases classified as Dent disease type 1 (DD1; OMIM #300009) [32], with more than 250 described mutations [37], reaching 524 unique variants identified in a recent study [38]. The *OCRL* gene is responsible for 15% of the cases classified as Dent disease type 2 (DD2; OMIM #300555) [39], with more than 140 described mutations [40]. The remaining 25% of cases correspond to clinically diagnosed Dent disease with no causal pathogenic variants identified after genetic testing [41]. DD2 patients also have additional extrarenal manifestations such as milder intellectual impairment, hypotonia, mild developmental delay, and sub-clinical cataract, which enable them to be distinguished from Lowe syndrome (OMIM #309000). This disorder is also caused by mutations in *OCRL* and is characterized by the presence of severe abnormalities in the eyes, the central nervous system, and the kidneys [42].

The exact prevalence of this pathology is unknown. According to nationwide registries performed by different countries, 62 patients have been identified in the Great Britain, 91 in Japan, and 108 in France. Therefore, a prevalence can be estimated between 1 in 400,000 and 1 in 1,000,000 individuals [35]. Specifically in Spain, approximately 30 cases of Dent disease-affected individuals have been described [43]. Most cited publications indicate that more than 250 families worldwide are affected by DD1 [44]. However, a recent study estimated that between 880 and 3520 families could be affected, based on the current number of reported *CLCN5* variants [38].

The *CLCN5* gene encodes the electrogenic 2Cl^−^/H^+^ antiporter ClC-5, which is expressed in the PT epithelial cells and in the intercalated cells of the collecting duct. This channel is predominantly located in the early endosomes from the subapical compartment, where it colocalizes with the electrogenic vacuolar H^+^-ATPase (V-ATPase) [45,46]. This channel works as a homodimer that spans the cell membrane, with each subunit having its own pore for selective transport [47,48]. Under physiological conditions, LMW proteins are filtered at the glomerulus and reabsorbed in the PT via the megalin/cubilin complex through receptor-mediated endocytosis. The acidification of early endosomes via V-ATPase results in the dissociation of ligands from the endocytic complex, to subsequently degrade the ligands in the lysosomes and recycle the endocytic receptor complex to the brush border membrane. The ClC-5 plays an important role in this process, as its absence causes a defective endocytosis of these molecules in the PT epithelial cells, associated with a complete or partial loss of the megalin/cubilin complex at the brush border membrane [48]. Also, the loss of ClC-5 functionality impairs ligand processing in the endosomes and lysosomes due to an impairment in vacuolar acidification [48].

The *OCRL* gene encodes an inositol polyphosphate 5-phosphatase expressed in the glomerulus, proximal tubules, and collecting duct and is primarily located in the plasma membrane, clathrin-coated vesicles, the trans-Golgi network, endosomes, and other cellular surface compartments [49]. This protein preferentially dephosphorylates the phosphatidyl-inositol 4,5-biphosphate (PI(4,5)P_2_), a highly abundant lipid in the plasma membrane, to regulate the membrane transport and cytoskeletal structure [42,50]. During the endocytic process, OCRL promotes the conversion of clathrin-coated vesicles with high PI(4,5)P_2_ levels into uncoated internalized vesicles characterized by lower PI(4,5)P_2_ levels. This protein is also associated with early endosomes, where low PI(4,5)P_2_ levels must be maintained to ensure proper polymerization of the actin skeleton [51]. This process enables vesicles trafficking toward the Golgi apparatus, degradation systems, and the plasma membrane for receptor recycling. Consequently, the loss of OCRL function impairs cellular endocytosis and intracellular vesicular trafficking, causing the accumulation of clathrin-coated vesicles and a defective receptor recycling to the plasma membrane, as well as the trapping of megalin in the early endosomes [51].

Dent disease is often diagnosed in advanced stages or sometimes remains undiagnosed, as proteinuria in children tends to be associated with glomerular lesions, therefore being misdiagnosed as nephrotic syndrome [52]. So, the diagnosis is currently focused on targeting the main symptoms such as LMW proteinuria, hypercalciuria, and progressive renal failure in male patients with a family history of renal diseases on the maternal side and without other manifestations such as hypoalbuminemia [52]. Genetic testing is recommended to confirm the clinical diagnosis of suspected patients and family members by identifying pathogenic variants in either *CLCN5* or *OCRL* [35].

Although no curative treatment is currently available for Dent disease, an early diagnosis has important management implications, as supportive measures may slow disease progression. Clinical management is focused on reducing hypercalciuria, preventing nephrocalcinosis and kidney stone formations, and delaying the evolution to CKD. The main interventions include adequate hydration and dietary modifications, such as limiting calcium, sodium, and oxalate intake [53]. Also, diuretic drugs, such as hydrochlorothiazide, can be administered to increase calcium reabsorption at the distal tubule [35]. Growth hormone therapy is administered in approximately 27% of DD1 patients and 54% of DD2 patients [54], specifically in those who develop growth impairment secondary to metabolic abnormalities such as vitamin D deficiency, hypophosphatemia and hypercalciuria.

## 3. Thick Ascending Limb of the Loop of Henle

### 3.1. Physiology and Molecular Basis of Disease

**Figure 2:**
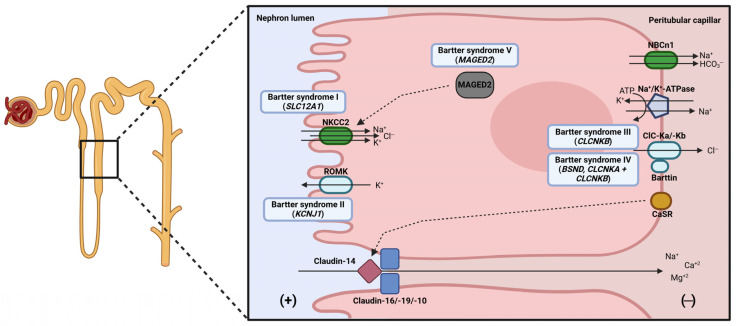
Main transport mechanisms from the epithelial cells of the TAL of the loop of Henle, with the causal genes of Bartter syndrome indicated as an example of tubulopathy affecting this nephron segment.

The loop of Henle is the segment of the nephron following the PT and it plays a key role in increasing tubular fluid concentration and preventing excessive water loss. Specifically, the descending limb reabsorbs water by osmosis thanks to the high permeability of this tubular segment, thereby increasing the concentration of the tubular fluid. Once in the ascending limb, ions are actively reabsorbed from the lumen, generating a hypotonic fluid which favors urine concentration in the collecting duct. For simplicity, the loop of Henle is discussed focusing on the thick ascending limb (TAL), which concentrates most of the active transport processes and is the primary site of disease-causing defects (Figure 2). The epithelial cells from the TAL are responsible for the residual reabsorption of 30% of filtered sodium and chloride, 25% of filtered calcium, 50–60% of magnesium, and 15% of bicarbonate [3,55].

In this segment, sodium, potassium, and chloride are reabsorbed from the lumen by the furosemide-sensitive cotransporter NKCC2 (encoded by *SLC12A1*), specifically expressed in this segment of the nephron [56]. This movement of ions happens as a response to the sodium concentration gradient generated by the Na^+^/K^+^-ATPase.

However, the potassium influx from both the luminal and basolateral regions is compensated by its secretion to the lumen via the renal outer medullary potassium (ROMK) channel (encoded by *KCNJ1*) located in the apical membrane [57]. So, the net reabsorption of potassium in the TAL is minimal, as 90% of ions absorbed by the NKCC2 are recycled back to the renal lumen, generating a lumen-positive transepithelial potential [55]. Loss-of-function mutations in *SLC12A1* and *ROMK* cause Bartter syndrome type I (OMIM #601678) and II (OMIM #241200), characterized by urinary salt loss and hypokalemic metabolic alkalosis, which is further developed in the following section [56,57].

Chloride effluxes from the cells via the anion-selective ClC-Kb and ClC-Ka chloride channels (encoded by *CLCNKB* and *CLCNKA*, respectively) following its concentration gradient [3]. These chloride channels are expressed in the basolateral membrane together with Barttin (encoded by *BSND*), a protein acting as a subunit needed for the proper functioning of the channels [58,59]. Mutations in ClC-Kb are responsible for Bartter syndrome type III (OMIM #607364) [60], while alterations in Barttin and digenic mutations in both ClC channels cause Bartter syndrome type IV (OMIM #302522, #313090) with slightly more severe symptoms and extrarenal manifestations [61,62].

As mentioned above, this segment of the nephron presents a lumen-positive transepithelial potential difference generated by potassium recycling back to the lumen via ROMK and the basolateral depolarization due to chloride efflux from the TAL epithelial cells [55]. Therefore, there is a paracellular cation movement derived from this electrochemical gradient. Calcium reabsorption in the TAL, representing 25% of the total filtered calcium, is carried out via paracellular transport dependent on specific claudins, as well as in the PT [3]. In this case, claudin-16 and claudin-19 (encoded by *CLDN16* and *CLDN19*, respectively) physically interact to generate the cation-selective pore located in the tight junctions of the TAL epithelial cells [63]. In addition, the movement of calcium cooccurs with the flux of magnesium ions, as the TAL reabsorbs 50-60% of the total filtered magnesium [55]. Another relevant member of this protein family is claudin-10 (encoded by *CLDN10*), which allows the paracellular flux of sodium through the TAL [64]. Mutations in *CLDN16* and *CLDN19* cause hypomagnesemia with hypercalciuria and nephrocalcinosis (OMIM #248250 and OMIM #248190, respectively) [65,66], while mutations in *CLDN10* are responsible for HELIX syndrome (OMIM #617671), characterized by hypermagnesemia, hypokalemic alkalosis, and salt wasting in urine [64,67].

The transport of ions in the TAL is regulated by several hormones that stimulate (vasopressin, PTH, angiotensin II, glucagon …) and inhibit (extracellular calcium via CaSR, prostaglandin E2, nitric oxide …) the transepithelial reabsorption of sodium and chloride [68]. For example, vasopressin acting via the V2-receptor (encoded by *AVPR2*) increases intracellular cAMP concentration, which in turn stimulates the exocytosis of NKCC2 cotransporter from the subapical vesicles to the apical membrane [69,70]. In contrast, in response to high serum calcium concentration, sensed by the basolateral CaSR receptor, the expression of claudin-14 (encoded by *CLDN14*) is induced, causing the inhibition of paracellular calcium reabsorption due to claudin-16-claudin-19 pore blockage [71]. Moreover, gain-of-function mutations in the calcium-sensing receptor CaSR cause a Bartter-like syndrome known as autosomal dominant hypocalcemia (OMIM #601198) [72].

TAL also participates in acid-base homeostasis as it reabsorbs the residual filtered bicarbonate and excretes ammonium in an NHE3 and carbonic anhydrase-dependent manner, which also happens in the PT [73]. Bicarbonate exits the cells via basolateral Cl^−^/HCO_3_^−^ and Na^+^/H^+^ exchangers and K^+^-HCO_3_^−^ cotransporter [74]. In addition, basolateral NBCn1 cotransporter also mediates bicarbonate flux thanks to sodium movement, although the direction of the flow is not fully understood [75,76]. Ammonium can be reabsorbed in the TAL mainly in response to acidosis through apical NKCC2 and other potassium channels [77], as ammonium has a similar ionic radius as potassium ions and effluxes from the cells through the basolateral NHE4 Na^+^/H^+^ exchanger [78].

Collectively, these molecular mechanisms highlight the functional complexity of the TAL, where both transcellular and paracellular transport systems contribute to electrolyte homeostasis. While alterations in tight-junction proteins such as claudins lead to selective defects in divalent cation handling, disturbances in transcellular NaCl transport result in a more global impairment of TAL function. In this context, Bartter syndrome represents the paradigmatic disorder of the TAL, as alterations in several transport mechanisms are responsible for this tubulopathy, and it is therefore discussed in detail below.

### 3.2. Bartter Syndrome

Bartter syndrome is a salt-wasting tubulopathy affecting the TAL of the loop of Henle, with an estimated prevalence of 1 per 1,000,000 individuals [79]. The main clinical manifestations are hypokalemia, metabolic alkalosis, hypercalciuria, low blood pressure, and hyperreninemic hyperaldosteronism. The symptomatology derives from a defective reabsorption of sodium chloride in the TAL, as in physiological conditions up to 30% of the total renal filtered salt is reabsorbed. Bartter syndrome also presents as an antenatal form characterized by a very acute and early onset, with a high risk of perinatal mortality. This form is caused by fetal polyuria leading to severe polyhydramnios between the 24th and 30th week of gestation, high salt wasting, and extreme prematurity [80].

This syndrome can be classified into six different subtypes according to the affected gene, most of which follow an autosomal recessive inheritance pattern, except for two subtypes with digenic and X-linked inheritance. Mutations causing Bartter syndrome are suggested to disrupt the lumen-positive transepithelial voltage in the TAL region, with a variable severity depending on the affected gene.

Bartter syndrome type I (OMIM #601678) is caused by mutations in the *SLC12A1* gene encoding the NKCC2 cotransporter [56]. This protein acts as the main mechanism of transepithelial NaCl reabsorption, regulating urine concentration and volume. Bartter syndrome type II (OMIM #241200) is caused by mutations in the *KCNJ1* gene encoding ROMK [57], which is needed for the proper functioning of NKCC2, explaining the similar prenatal presentation of Bartter syndrome type I and II. These two forms of Bartter syndrome, also known as antenatal Bartter syndrome, are the most severe with a very early onset and course with severe polyhydramnios, premature birth, calcium wasting and nephrocalcinosis.

Bartter syndrome type III (OMIM #607364) is caused by mutations in the *CLCNKB* gene encoding the Cl^−^ voltage-gated channel ClC-Kb, leading to a defective reabsorption of Cl^−^ at the TAL [60,61]. This form is also known as classical Bartter syndrome, which is less severe and courses with a greater phenotypical variability between patients [60]. Bartter syndrome type IV is caused by mutations in the *BSND* gene (Bartter syndrome type IVa; OMIM #302522), the accessory subunit of the CLC-Ka and CLC-Kb [61], and digenic mutations in both *CLCNKA* and *CLCNKB* genes (Bartter syndrome type IVb; OMIM #313090) [62]. These proteins are located in the TAL and in the stria vascularis of the inner ear. Therefore, Bartter syndrome type IV is associated with sensorineural deafness due to the loss of potential load in the inner ear.

Bartter syndrome type V (OMIM #300971) is an X-linked disease subtype caused by mutations in the *MAGED2* gene, which has an important role in both developing and adult kidneys [81]. This gene is expressed in the TAL and the distal tubules, where it has a role in the maintenance of amniotic fluid homeostasis and fetal renal salt reabsorption. This subtype is characterized by its very acute and early onset as fetal kidneys are the predominant source of amniotic fluid. Mutations in *MAGED2* affect the expression of NKCC2 and NCC, both sodium chloride cotransporters expressed in the fetal tubular cells. In physiological conditions, MAGED2 may regulate membrane protein maturation in the endoplasmic reticulum and increase apical expression of NKCC2 and NCC transporters. However, in contrast to Bartter syndrome type I and II, this antenatal form resolves spontaneously in childhood [81].

The diagnosis of Bartter syndrome is based on the evaluation of symptoms—such as renal salt wasting, signs of dehydration, and polyuria—and biochemical alterations, including electrolyte imbalance with hypokalemia, hypercalciuria, and, in some cases, hypomagnesemia, together with high renin-aldosterone blood concentration [82]. Polyhydramnios and prematurity are the key clinical manifestations for the diagnosis of antenatal Bartter syndrome. Clinical suspicions can be confirmed with genetic testing to identify mutations in the *SLC12A1*, *KCNJ1*, *CLCNKB*, *CLCNKA*, *BSND*, and *MAGED2* genes, which also help in resolving complex cases with overlapping phenotypes [80].

Current treatment strategies rely on liberal dietary salt intake and oral potassium and magnesium supplementation to restore electrolyte levels, requiring continuous monitoring due to the chronic nature of the disorder. In addition, nonsteroidal anti-inflammatory drugs, potassium-sparing diuretics, angiotensin-converting-enzyme inhibitors, and angiotensin receptor blockers can be used to ameliorate hypokalemic metabolic alkalosis and secondary hyperaldosteronism [82]. In cases of polyhydramnios, maternal treatment with nonsteroidal anti-inflammatory drugs is considered along with close monitoring of the fetus.

## 4. Distal Convoluted Tubule

### 4.1. Physiology and Molecular Basis of Disease

**Figure 3:**
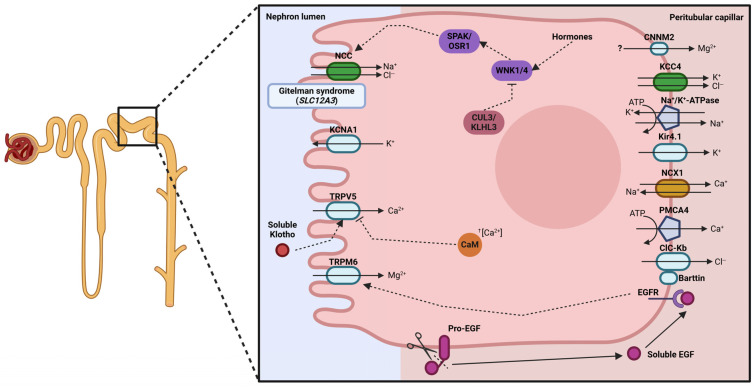
Main transport mechanisms from the DCT epithelial cells, with the causal gene of Gitelman syndrome indicated as an example of tubulopathy affecting this nephron segment.

The distal convoluted tubule (DCT) plays a crucial role in the fine-tuning of electrolyte homeostasis, acting as a major regulatory site for sodium, chloride, calcium, and magnesium reabsorption after the bulk transport processes of the upstream nephron segments (Figure 3). Unlike proximal nephron segments, the DCT is characterized by low water permeability, which allows precise hormone-regulated electrolyte handling without water reabsorption, an essential process for maintaining systemic mineral balance.

DCT is characterized by the expression of the thiazide-sensitive NaCl cotransporter NCC (encoded by *SLC12A3*) [83], which reabsorbs the remaining 5–10% of filtered sodium together with chloride ions [84]. Loss-of-function mutations in *SLC12A3* cause Gitelman syndrome (OMIM #263800), a salt-wasting tubulopathy that will be further discussed in the following section [85]. Moreover, the active form of NCC depends on its phosphorylation by the 2 serine-threonine kinases SPAK and OSR1, which increase the Na^+^/Cl^−^-reabsorptive properties of NCC [86]. The SPAK/OSR1 complex and the NCC transporter are, in turn, regulated by the WNK1 and WNK4 kinases and their degradation protein complex CUL3/KLHL3. This regulative complex is important as such pathogenic variants in either of these four proteins cause different genetic forms of pseudohypoaldosteronism type 2 (OMIM #614491, #614492, #614495, and #614496) due to NCC overactivity [87,88].

Specifically, WNK4 inhibits NCC trafficking from the intracellular storage pool to the apical membrane [89], a process that can be reversed by WNK1 [90]. Also, both kinases can phosphorylate SPAK/OSR1, which in turn stimulates NCC activity [86]. In contrast, KLHL3 favors the binding of the ubiquitin ligase CUL3 to WNKs, resulting in their degradation [91]. Therefore, mutations in WNK4 avoid the binding to KLHL3, leading to an increased WNK4 expression [92]. Also, this leads to an increased stimulation of the SPAK/OSR1 [93] and a reduction in the inhibitory effect on NCC trafficking [94]. So, mutations in WNK4 cause an increased expression of NCC in the apical membrane and a stimulation of the channel activity via SPAK/OSR1. In turn, mutations in WNK1 increase total protein expression, avoiding KLHL3/CUL3-mediated degradation, and enhance the stimulation of NCC activity through SPAK/OSR1-increased phosphorylation [87,95]. In addition, an increased activity of WNK1 leads to a greater inhibition of WNK4, favoring the trafficking of NCC to the apical membrane [90]. Finally, mutations in KLHL3 and CUL3 cause a reduced degradation of both WNKs, leading to NCC overactivity via increased vesicular trafficking and phosphorylation [88,91,92]. NCC activity can also be stimulated by different hormones, such as vasopressin [96], aldosterone [97], angiotensin II [98], and insulin [99], acting via the SPAK/OSR1 and WNKs pathway.

As in other nephron segments, transcellular sodium movement driven by the Na^+^/K^+^-ATPase-generated gradient requires basolateral potassium recycling [100]. An important potassium channel in the DCT is the Kir4.1 (encoded by *KCNJ10*), as loss-of-function mutations lead to the EAST/SeSAME syndrome (OMIM #602208), characterized by renal salt loss and neurologic abnormalities due to an indirect impairment of the Na^+^/K^+^-ATPase and, consequently, a reduced sodium uptake [101,102].

The reabsorption of sodium from the luminal space depends on the flux of chloride through the NCC cotransporter. This movement depends on the gradient generated by the efflux of chloride ions to the basolateral region. Therefore, via the negative potential difference generated by the Na^+^/K^+^-ATPase, chloride ions efflux the DCT cells through the ClC-Kb/Barttin complex, which happens in the TAL [58,59]. In addition, low intracellular chloride concentration maintains the NCC cotransporter in an active state [103]. Moreover, chloride, together with potassium, effluxes from the cells via the basolateral KCC4 cotransporter (encoded by *SLC12A7*) following the electrochemical gradient [104].

The remaining 3–7% of filtered calcium is actively reabsorbed in the DCT, in contrast to other nephron segments, where calcium uptake happens mainly via paracellular mechanisms [3]. So, this transcellular reabsorption process is predominantly dependent on the apical epithelial calcium channel TRPV5 (encoded by *TRPV5*) [105]. This receptor is regulated by molecules and hormones such as 1,25-dihydroxyvitamin D3 and PTH, which promote TRPV5 transcription and avoid its degradation, resulting in an increment in calcium uptake [106,107]. In addition, the protein Klotho, via its capacity to hydrolyze extracellular sugar residues from other proteins, increases the residence time of TRPV5 in the apical plasma membrane, avoiding its degradation and favoring calcium uptake [108]. In contrast, in high intracellular calcium conditions, the calcium-sensing protein calmodulin inactivates TRPV5 by modulating its intracellular trafficking leading to its retention in the endoplasmic reticulum and decreasing its plasma membrane abundance [109]. At the basolateral membrane, the calcium ATPase PMCA4 and the Na^+^/Ca^2+^ exchanger NCX1 (encoded by *SLC8A1*) mediate the calcium flux from the intracellular space [106,110].

As happens with the calcium movement, the residual 10% of filtered magnesium ions are reabsorbed in this part of the nephron via active apical transcellular mechanisms. The key player of this process is the transient receptor potential cation channel subfamily M member 6 (encoded by *TRPM6*), a voltage-driven cation channel whose transport activity depends on the potential generated by the Na^+^/K^+^-ATPase [111]. Therefore, loss-of-function mutations in this channel lead to a magnesium-wasting disorder known as hypomagnesemia type 1 with secondary hypocalcemia (OMIM #602014) [111]. Also, soluble EGF has been described as a magnesium reabsorption regulator via its binding to its specific receptor, which activates a signaling pathway to stimulate TRPM6 [112]. Therefore, mutations in the basolateral membrane protein pro-EGF expressed in the DCT cells cause hypomagnesemia type 4 (OMIM #611718), as an inefficient location of the protein leads to an inadequate cleavage and release of soluble EGF [113].

In addition, mutations in the α1 (encoded by *ATP1A1*) and γ (encoded by *FXYD2*) subunits of the Na^+^/K^+^-ATPase also lead to magnesium-wasting diseases, such as hypomagnesemia with seizures and intellectual disability (OMIM #618314) [114] and autosomal dominant primary hypomagnesemia hypocalciuria (OMIM #154020) [115]. Moreover, loss-of-function mutations in apical Kv1.1 (encoded by *KCNA1*) and basolateral Kir4.1 (encoded by *KCNJ10*) potassium channels also alter the movement of magnesium ions, leading to autosomal dominant hypomagnesemia (OMIM #160120) [116] and explaining the magnesium wasting observed in the above-mentioned EAST/SeSAME syndrome (OMIM #602208) [102].

In contrast, basolateral magnesium efflux is currently not fully understood, although some proteins have been proposed to participate in this process. For example, the basolateral expressed protein Cyclin M2 (encoded by *CNNM2*) has been proposed as a possible basolateral magnesium/divalent cations transporter [117]. However, other studies suggest that Cyclin M2 works as an intracellular magnesium sensor that could regulate the reabsorption of magnesium in the kidney [118]. Independent of Cyclin M2 mechanism of action, it is known that loss-of-function mutations in *CNNM2* cause hypomagnesemia and other symptoms associated with low magnesium, such as seizures and intellectual disabilities (OMIM #616418) [119].

Therefore, the physiology and molecular basis of DCT diseases allow the understanding of how alterations in ion transport mechanisms result in characteristic hydroelectrolytic imbalances. This context serves as a starting point for addressing Gitelman syndrome, the most prevalent rare tubulopathy among all.

### 4.2. Gitelman Syndrome

Gitelman syndrome (OMIM #263800) is an autosomal recessive salt-wasting tubulopathy affecting the DCT and is characterized by hypokalemic metabolic alkalosis, hypomagnesemia and hypocalciuria, typically present in late adolescence or adulthood [120]. The estimated prevalence ranges between 1 and 10 per 40,000 people worldwide, making it the most frequent inherited tubulopathy [121]. The main clinical manifestations are fatigue, dizziness, chondrocalcinosis, seizures, muscle weakness, and ventricular arrhythmia, among others, which impair patients’ quality of life, although in some cases only mild symptoms are reported [122].

More than 350 inactivating mutations in the *SLC12A3* gene, encoding the NCC cotransporter, have been reported to cause Gitelman syndrome [85,122]. NCC is expressed in the apical membrane of the DCT epithelial cells, where it reabsorbs sodium and chloride from the urine [85]. Mutations in *SLC12A3* lead to impaired NCC synthesis or to protein misfolding, resulting in endoplasmic reticulum retention and subsequent proteasomal degradation [123].

Therefore, NCC-deficient cells exhibit reduced Na^+^/Cl^−^ reabsorption in the DCT, leading to increased sodium concentration in the collecting duct (CD), which results in mild volume contraction and stimulation of the renin–angiotensin–aldosterone cascade [122]. First, the extracellular volume contraction leads to an increase in the paracellular calcium reabsorption with unaltered active calcium transport, causing hypocalciuria [124,125]. As will be reviewed in the following sections, hyperaldosteronism causes an increase in sodium reabsorption in the CD via the ENaC channel, which in turn enhances potassium and hydrogen secretion, resulting in hypokalemic metabolic alkalosis. In addition, reduced abundance of the TRMP6 magnesium channel at the apical membrane of the DCT epithelial cells leads to reduced magnesium uptake, thereby explaining the associated hypomagnesemia [125].

The diagnosis of Gitelman syndrome is very similar to Bartter syndrome. Some differential clinical features of Gitelman syndrome are hypomagnesemia and hypocalciuria, which are diagnosed during late childhood or adulthood. However, between Gitelman and Bartter syndrome type III there are overlapping phenotypes, making differential diagnosis based solely on clinical features difficult [82,122]. In addition, pathogenic variants in other genes, such as *HNF1β* can lead to similar electrolytic imbalances [126]. Therefore, the clinical suspicion has to be confirmed via genetic testing to detect the biallelic inactivating mutations in *SLC12A3*. The current treatments are also similar to the ones administered to Bartter syndrome patients, based on the restoration of electrolyte levels and the improvement of symptomatology, by administering potassium and magnesium supplementation and following dietary recommendations [82].

## 5. Collecting Duct

### 5.1. Physiology and Molecular Basis of Disease

**Figure 4:**
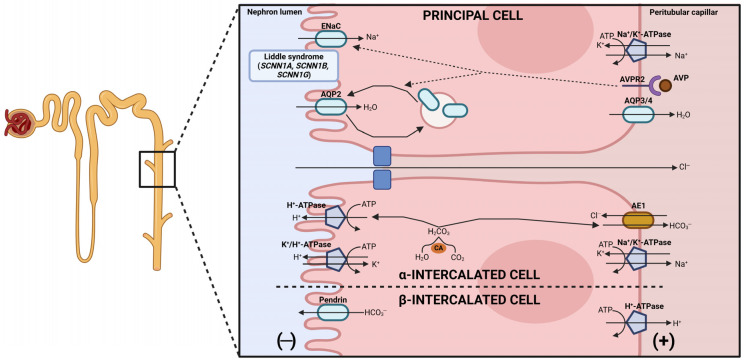
Main transport mechanisms from the different epithelial cells of the CD, with the causal genes of Liddle syndrome indicated as an example of tubulopathy affecting this nephron segment.

The collecting duct (CD) connects the nephron to the ureter and is key for the final regulation of water, acid-base, and electrolyte homeostasis, determining the final composition of urine. This segment integrates hormonal signaling, such as vasopressin and aldosterone, to finely adjust tubular reabsorption and secretion according to systemic needs. As reviewed later, in the absence of vasopressin, the CD is largely impermeable to water, allowing urine dilution, whereas vasopressin promotes water reabsorption. Within this segment, it is important to distinguish between the different cell types present in the CD (Figure 4).

Firstly, principal cells are characterized by the expression of the apical epithelial sodium channel (ENaC) and aquaporin-2 (encoded by *AQP2*) and the basolateral aquaporin-3 and aquaporin-4 (encoded by *AQP3* and *AQP4*, respectively) [127]. Therefore, the main function of these cells is the maintenance of water and sodium homeostasis. In contrast, intercalated cells, which regulate acid-base balance, express the vacuolar-type H^+^-ATPase (V-ATPase) and the carbonic anhydrase II (CAII) [128].

Intercalated cells can be classified into two distinct subtypes: α-intercalated cells and β-intercalated cells. The first subgroup is characterized by the apical expression of the V-ATPase and the basolateral expression of the anion exchanger 1 (AE1, encoded by *SLC4A1*) [129]. So, α-intercalated cells secrete protons to the lumen and reabsorb filtered bicarbonate in exchange for chloride, resulting in urine acidification. In contrast, β-intercalated cells express the V-ATPase in the basolateral membrane to reabsorb protons and secrete bicarbonate to the lumen via the apical pendrin channel (encoded by *SLC26A4*) [129,130].

The sodium channel ENaC of the principal cells is composed of the α, β, and γ homologous subunits (encoded by *SCNN1A*, *SCNN1B*, and *SCNN1G*, respectively) [131]. In contrast to the PT, the TAL of the loop of Henle and the DCT, sodium reabsorption is not directly associated with chloride uptake, as ENaC only acts on sodium following its own transepithelial gradient. Alterations in the genes encoding for the ENaC subunits have to be differentiated between gain-of-function and loss-of-function mutations, as each of them is associated with a different pathology. Pseudohypoaldosteronism type 1B (OMIM #264350, #620125, and #620126) is caused by loss-of-function mutations in either of the three ENaC subunits. This disorder is characterized by high sodium wasting leading to hyponatremia, hyperkalemia, hyperreninemia, hypotension, and high serum aldosterone concentration [132]. In contrast, gain-of-function mutations in ENaC cause Liddle syndrome (OMIM #618126, #177200, and #618114), which courses with a symptomatology opposite to that of pseudohypoaldosteronism type 1B [133], as discussed in the following section. At a different regulatory level of the same aldosterone—ENaC signaling axis, pseudohypoaldosteronism type 1A (OMIM #177735) is caused by loss-of-function mutations in the mineralocorticoid receptor (encoded by *NR3C2*) leading to a non-responsive situation to aldosterone, although having high serum levels of this hormone [134].

Chloride mainly moves paracellularly through specific claudins, such as claudin-3, -4, -7 and -8 (encoded by *CLDN3*, *CLDN4*, *CLDN7* and *CLDN8*, respectively), thanks to the lumen-negative transepithelial potential generated by the sodium movement [135]. As mentioned above, chloride uptake also depends on the movement of bicarbonate via Cl^−^/HCO_3_^−^ exchangers as AE1, pendrin, AE4 (encoded by *SLC4A4*), *SLC26A11* and *SLC26A7* [128]. Acid-base homeostasis regulation is a key process in the CD, as dysfunctions in the α-intercalated cells lead to impaired urinary acidification, favoring the apparition of renal lithiasis, low plasma bicarbonate levels and bone manifestations. This set of clinical manifestations is known as distal renal tubular acidosis (OMIM #611590, #267300, #602722) and is caused by mutations in several proteins involved in the machinery responsible for urinary acidification [136].

First, loss-of-function mutations in the chloride/bicarbonate exchanger AE1 avoid the reabsorption of bicarbonate from the renal lumen [137,138]. Mutations in the B1 and A4 subunits from the proton V-ATPase (encoded by *ATP6V1B1* and *ATP6V0A4*, respectively) cause an inefficient urine acidification due to the inability of the cells to secrete protons to the renal lumen [139]. In addition, alterations in the transcription factor FOXI1 lead to a decreased expression of the above-mentioned genes (*ATP6V1B1*, *ATP6V0A4*, *SLC4A1*, *SLC4A4*, and *SLC26A4*), all of them being key players in the acid-base homeostasis [140]. Lastly, distal renal tubular acidosis is also due to mutations in *WDR72*, which is involved in vesicle transport, microtubular assembly, and membrane mobilization [136]. Therefore, dysfunctions in WDR72 can cause intracellular retention of these key players [136].

As in earlier nephron segments, potassium secretion by principal cells is essential for maintaining the sodium gradient, a process driven by the Na^+^/K^+^-ATPase, thereby leading to potassium efflux into the tubular lumen through specific apical channels [141]. However, under conditions of hypokalemia, potassium can be reabsorbed via the apical H^+^/K^+^-ATPase expressed in α-intercalated cells, allowing potassium conservation in exchange for proton secretion [142].

The CD is the most important renal segment for water homeostasis, as it has a higher water permeability than earlier nephron segments. Water is reabsorbed following the osmotic gradient in principal cells through the apical aquaporin-2 channel, which under basal conditions is mainly stored in a subapical intracellular vesicle pool [143]. Water then exits the cells via the aquaporin-3 and aquaporin-4 channels located in the basolateral membrane [144,145].

The function of the CD is highly regulated by the antidiuretic hormone vasopressin and the steroid hormone aldosterone, which increase the osmotic gradient for water reabsorption, acting through electrolyte channels, and favoring transcellular water transport via specific channels. Specifically, vasopressin acting through the basolateral V2-receptor (encoded by *AVPR2*) increases ENaC surface expression by stimulating exocytosis from the intracellular vesicle pool, reducing channel endocytosis and degradation, and increasing ENaC open probability [146]. In addition, vasopressin increases *AQP2* gene transcription and apical membrane expression, acting on the intracellular vesicle pool as happens with ENaC, which in turn increases water permeability in the CD [146]. Aldosterone acts in a similar way as vasopressin, increasing ENaC activity, favoring aquaporin-2 surface expression, and stimulating the transcription of *AVPR2* [147]. Therefore, the defects in the regulation of water reabsorption led to an inefficient capacity of urine concentration and polyuria, as happens in patients with mutations in *AQP2* or *AVPR2*, causing nephrogenic diabetes insipidus (OMIM #304800 and #125800) [148,149].

In this context, Liddle syndrome is further developed in the following section as an example of a disease caused by inappropriate gain-of-function of a transport mechanism. Notably, this disorder is one of the few hereditary tubulopathies for which an effective targeted pharmacological treatment is available.

### 5.2. Liddle Syndrome

Liddle syndrome, also known as pseudohyperaldosteronism, is a very rare autosomal dominant tubulopathy from the CD with an unknown current prevalence. The main clinical manifestations are early-onset hypertension, hypokalemic metabolic alkalosis, hyporeninaemia, and hypoaldosteronism [133]. This symptomatology can be explained by an increased sodium reabsorption in the CD via the aldosterone-regulated amiloride-sensitive sodium channel ENaC.

As mentioned above, Liddle syndrome is caused by gain-of-function mutations in the *SCNN1A* (OMIM #618126), *SCNN1B* (OMIM #177200) and *SCNN1G* (OMIM #618114) genes that encode the α, β and γ subunits of ENaC, respectively [150,151,152]. Each subunit contains two transmembrane domains, extracellular loop, and short intracellular N- and C-terminal regions [153]. The highly conserved C-terminal region plays a critical role in Liddle syndrome pathogenesis, as its loss in the β and γ subunits causes an increased ENaC currents [133,150]. However, this gain-of-function does not arise from altered biophysical properties of the channel, such as an increased conductance or open probability. Instead, ENaC hyperactivity is driven by increased surface expression at the apical membrane, due to impaired internalization and degradation mediated by the Nedd4-2 ubiquitin-proteasome system [154,155].

In contrast, mutations in the α subunit have been shown to increase ENaC activity by enhancing channel currents without changes in surface expression [152]. Consequently, the increased ENaC activity or abundance leads to enhanced sodium reabsorption independently of aldosterone regulation. This sodium uptake, accompanied by increased water reabsorption and potassium excretion, ultimately results in the development of hypertension.

The diagnosis of Liddle syndrome is based on the identification of characteristic clinical features, including hypertension associated with hypokalemic alkalosis and impaired renin and aldosterone levels. Genetic testing should be considered in patients with early-onset treatment-resistant hypertension in the context of low plasma renin and aldosterone concentrations. Therefore, diagnosis of Liddle syndrome can be confirmed by the identification of pathogenic variants in *SCNN1A*, *SCNN1B*, and *SCNN1G*. This disorder has a specific and effective treatment based on the administration of potassium-sparing diuretics, such as amiloride or triamterene, which correct the hypokalemic metabolic alkalosis and elevated blood pressure [156]. These drugs act as ENaC antagonists, inhibiting sodium reabsorption and thereby preventing potassium excretion via Na^+^/K^+^ exchangers. Therefore, accurate diagnosis of Liddle syndrome is crucial to enable appropriate treatment, avoid complications, and achieve a favorable prognosis.

## 6. Current Challenges and Future Perspectives

### 6.1. Limitations and Novel Opportunities in Diagnosis

In this review, we have highlighted the importance of the proper functioning of the different transport systems across all nephron segments and the significant impact that alterations in these molecular pathways have on renal tubular physiology. Despite the substantial advances in molecular genetics, the specific monogenic cause remains unknown in 50–60% of cases of inherited tubulopathies [157]. This diagnostic gap can be attributed to the small size of patients’ cohorts, the lack of knowledge of novel disease-causing genes, the incomplete penetrance of some pathogenic variants, and the unknown functional consequence of variants of unknown significance, all of which limit the diagnostic yield of genetic testing. In addition, technical limitations complicate the identification of non-coding variants and complex genomic rearrangements, which also contribute to the proportion of unresolved cases.

For example, genetic testing in Gitelman syndrome aims to identify the biallelic inactivating mutations in *SLC12A3*. It should be noted that routine genetic screening strategies should also include analysis for the identification of copy number variations. Using this combined approach, pathogenic variants in both *SLC12A3* alleles have been identified in approximately 80–85% of patients diagnosed with Gitelman syndrome [158]. However, between 18 and 40% of patients with a clinical diagnosis, only one mutation is identified after standard genetic screening [159]. This finding may reflect the presence of a second undetected pathogenic allele, including regulatory or deep intronic variants that lead to aberrant mRNA splicing. Such variants are not captured by conventional sequencing assays, highlighting the limitations of current genetic testing approaches. Consequently, advances in genetic testing tools, together with a growing knowledge of the human genome, are expected to facilitate the identification of novel variants and disease-causing genes, enabling a more precise diagnosis of the affected patients.

In addition, these disorders exhibit marked heterogeneity in terms of clinical presentation, disease progression, age of onset, and prevalence across different populations, with an unknown genotype-phenotype correlation. Dent disease is an example of a disorder with an unclear genotype-phenotype correlation. Clinical differences between DD1 and DD2 are explained by the broader expression of *OCLR* in different tissues, thereby leading to extrarenal manifestations in the brain and the eyes, while *CLCN5* is mainly expressed in the kidney. However, some patients carrying pathogenic variants in either *CLCN5* or *OCRL* show incomplete penetrance. Illustrating this variability, cases have been reported in which brothers carrying the same mutation in *CLCN5* or *OCLR* present with markedly different clinical phenotypes [160,161]. For example, one brother exhibited proteinuria, hypercalciuria, and nephrocalcinosis, whereas the other remained asymptomatic with normal biochemical parameters [162]. The mechanisms underlying this phenotypical variability are still unknown; however, some studies have suggested the involvement of modifier genes, such as *CFTR*, *SCNN1A*, and *SCNN1B*. The association is supported by the identification of heterozygous variants in these genes, classically associated with cystic fibrosis and cystic fibrosis-like diseases, in patients with Dent disease [163].

Focusing on *OCRL*-related disorders, DD2 has traditionally been considered a milder variant of Lowe syndrome. Several studies have attempted to establish genotype-phenotype correlation, reporting that most mutations located in exons 8–23 of *OCLR* are associated with Lowe syndrome, whereas mutations in exons 1–7 are linked to DD2 [164]. However, some mutations affecting the catalytic domain of *OCRL* have been identified in patients with either Lowe syndrome or DD2 [161]. Therefore, patients with the same pathogenic variant exhibit marked phenotypic heterogeneity or are even classified as having different clinical entities, suggesting the influence of modifying factors or genetic background. This lack of genotype-phenotype correlation complicates the diagnosis and management of patients with pathogenic *OCRL* variants.

Another example of diagnostic challenges involves variants in *HNF1β*, which can lead to a complex diagnostic situation. Affected patients may present with electrolyte imbalances such as hyperuricemia, hypocalciuria, and hypomagnesemia, which can be misdiagnosed as tubular disorders such as Gitelman syndrome. The reason behind this phenotypic complexity is the broad regulatory scope of *HNF1β*, as this transcription factor acts over the expression of many ion transport mechanisms in the kidney [126]. Among the *HNF1β*-regulated genes, we find several of them associated with kidney disorders, such as Bartter syndrome (*SLC12A1* and *KCNJ1*), Imerslund-Grasbeck syndrome (*CUBN*), hypomagnesemia (*FXYD2*), hypocalcemia (*CASR*), hypouricemia (*SLC22A12*), and distal renal tubular acidosis (*ATP6V1B1*). Therefore, these aspects pose a major challenge for accurate and early diagnosis, as well as optimal management of patients.

For this reason, the identification and validation of novel and specific biomarkers is a promising opportunity to support accurate diagnosis, improve kidney function assessment and enable effective monitoring of disease progression. While serum creatinine and proteinuria remain the gold standards in clinical practice, emerging approaches, such as the use of urinary extracellular vesicles offer a novel source of potential non-invasive biomarkers. For example, recent studies in Gitelman syndrome identified a characteristic expression pattern of renal tubular transporters in urinary extracellular vesicles, including NCC, NHE3, NKCC2, ENaCβ, pendrin, and ROMK [165]. In addition, these vesicles have also enabled the identification of differentially expressed miRNAs, such as the upregulation of hsa-let-7d-3p in Gitelman syndrome, which negatively regulates NEDD4L in the CD [166].

Therefore, these discoveries highlight the potential of urinary extracellular vesicles as a non-invasive source for biomarker identification and a tool to gain mechanistic insights into tubular disorders. Accordingly, this promising approach is being applied to the obtention of candidate biomarkers in other inherited renal tubulopathies to improve differential diagnosis, including Dent disease [167] and Bartter syndrome [168].

However, the diagnosis of rare inherited tubulopathies remains a major challenge, as overlapping clinical manifestations, phenotypic heterogeneity, and limited awareness of these renal disorders lead to incorrect or delayed diagnosis. As discussed throughout this review, these disorders may initially present with nonspecific clinical manifestations, such as electrolyte imbalances, which can be erroneously attributed to other renal or systemic conditions. From a clinical perspective, early identification of inheritance patterns and characteristic biochemical alterations is essential for accurate diagnosis and management of patients. However, limited standardized diagnostic algorithms and access to specialized genetic and biochemical testing—such as the assessment of LWM proteinuria in suspected cases of Dent disease—are important limitations in routine clinical practice. Therefore, the integration of genetic testing and novel biomarkers into diagnostic workflow for suspected hereditary tubulopathies may improve decision-making, avoid unnecessary treatments, and enhance long-term patient management.

### 6.2. Treatment Challenges and Emerging Therapeutic Strategies

As previously mentioned in this review, specific treatments are available for only a limited number of tubulopathies, highlighting the need for the development of novel therapeutic approaches to address currently untreatable conditions. For instance, Liddle syndrome has a well-established targeted therapy, as patients benefit from potassium-sparing diuretics, which significantly improve prognosis and clinical outcomes [156]. Another example of a tubulopathy with a targeted therapy is nephropathic cystinosis (OMIM #219800), caused by defects in the lysosomal cystine/H^+^ cotransporter cystinosin (encoded by *CTNS*) [169]. This disorder is characterized by lysosomal accumulation of cystine crystals, leading to progressive kidney damage. Accordingly, patients are treated with cysteamine, which binds to cystine forming a dimer that can be exported from lysosomes via the cationic amino acid transporter PQLC2 (encoded by *SLC66A1*) [170].

Other disorders show limited adherence to their designated pharmacological treatments or require careful patient-specific evaluation to approve their administration. Cystinuria is a clear example, as pharmacological treatment is only considered when conservative measures—such as dietary recommendations, hydration therapy, and urine alkalinization—are not sufficient to prevent stone formation and preserve renal function [171]. The restricted use of cystine-chelating agents, such as D-penicillamine and tiopronin, despite their proven efficacy in reducing cystine crystallization and dissolving stones, is due to severe adverse effects such as glomerulonephritis, nephrotic syndrome, hepatic abnormalities and mucocutaneous lesions [171,172]. Therefore, current studies focus on the evaluation of novel therapeutic approaches with improved efficacy and reduced toxicity, such as alternative cystine-chelating agents, such as bucillamine (NCT02942420), diuretic drugs such as tolvaptan [173] and antioxidant molecules such as α-lipoic acid [174] or L-Ergothioneine [175,176]. However, for most tubulopathies, as seen in Dent disease and Bartter and Gitelman syndromes, available treatments remain largely symptomatic, only addressing the consequences of the underlying molecular alterations but lacking effective and curative therapeutic strategies.

Nevertheless, recent studies have focused on the development of novel therapeutic strategies based on emerging techniques. Taking DD1 as an example, recent research has investigated the application of gene therapy by assessing the effects of lentiviral vector-mediated human *CLCN5* cDNA supplementation in ClC-5-null mice [177]. This mice model recapitulates the clinical phenotype of DD1, including diuresis, proteinuria, and hypercalciuria, making it suitable for evaluating the potential rescue of these altered parameters by using this gene-based therapy. After one and two months of treatment administration, an improvement in the disease was observed, as assessed by a reduction in LMW proteinuria. However, proteinuria and urine excretion values did not fully normalize to those of wild-type mice. Notably, the therapeutic effects were transient and disappeared four months after treatment, evidenced by the reappearance of proteinuria and increased diuresis. This loss of therapeutic effect was hypothesized to result from an activation of immune system-mediated responses against the transgene.

To overcome this limitation, in a following publication, tubule-specific promoters were used to avoid exogenous ClC-5 expression in dendritic cells [178]. Although treated kidneys showed successful expression of ClC-5 in the proximal tubules, no phenotypic improvement was observed in treated mice. In parallel, the same gene therapy from the previous publication was studied in newborn ClC-5-null mice, whose immune system is not fully developed. In this setting, a long-term reduction in LMW proteinuria was achieved, consistent with sustained expression of the *CLCN5* transgene. These findings suggest that effective and sustained gene therapy for DD1 may require transgene delivery before immune system maturation and that patients with DD1 may benefit from early-life therapeutic intervention.

Another promising approach is drug repurposing, as illustrated by studies exploring the potential use of alpelisib—a drug currently approved for clinical use in cancer therapy—in a mouse model of DD2 and Lowe syndrome [179]. As mentioned above, mutations in *OCRL* lead to increased levels of PI(4,5)P_2_, which are associated with aberrant actin polymerization and consequent impairment of endosomal trafficking. Based on this fact, the study hypothesized that phosphoinositide 3-kinase inhibitors such as alpelisib could rebalance phosphoinositide signaling and, thereby, rescue endocytosis. The results showed that treatment with alpelisib rescued the phenotype in the mouse model of DD2 and Lowe syndrome, as evidenced by improved endocytosis of LMW proteins. Additionally, a phase II pilot study is currently being performed to study the safety and efficacy of alpelisib in patients with DD2 (EUCT number: 2024-514196-17-00).

In addition, a drug approved for the treatment of urea cycle disorders, 4-phenylbutyrate (4PBA), has been proposed as a potential therapy for DD1 [180,181]. This rationale is supported by the ability of small molecules to restore protein folding, acting as chemical chaperones. Indeed, studies have shown that certain types of *CLCN5* mutations can be functionally rescued, as evidenced by improved Cl^−^ conductance in transfected cells expressing mutant *CLCN5* [181]. Moreover, 4PBA has also been evaluated in a DD1 mouse model, where treatment resulted in reduced urinary excretion of the LMW protein β2-microglobulin, although no improvements were observed in diuresis, calciuria and phosphaturia [180]. Therefore, further studies are required to assess the efficacy and safety of these therapeutic strategies in order to be considered for clinical application in patients with inherited renal tubulopathies.

Overall, current management of rare inherited tubulopathies remains supportive for the vast majority of cases and is further challenged by the diagnosis difficulties and limitations discussed above, together with the limited therapeutic options and the lack of curative strategies. Current approaches focus on correcting electrolyte imbalances, preventing complications and slowing disease progression, thereby highlighting the importance of early and accurate diagnosis for optimizing patient management. Therefore, emerging therapeutic strategies, such as targeted pharmacological interventions and gene-based therapies, together with the advances in the molecular characterization of tubular transport mechanisms, offer promising opportunities to overcome current limitations.

## 7. Conclusions

Taken together, future perspectives should focus on expanding genetic diagnostics, integrating multi-omics approaches and developing physiologically relevant cellular and animal models, in order to characterize candidate disease genes, improve the knowledge of these disorders, obtain novel biomarkers and develop new therapeutic approaches. Precision medicine tools, including genotype–phenotype correlations and new targeted molecular therapies, are essential to progress toward individualized, effective and curative treatment which will improve the quality of life of the patients. As our understanding continues to grow, bridging the gap between molecular knowledge and clinical application is crucial for improving outcomes for patients with rare inherited renal tubulopathies.

## Figures and Tables

**Table 1:** List of rare inherited renal tubulopathies.

Disorder	Inheritance	Gene	Protein	Protein Function	OMIM
PROXIMAL TUBULE					
Dent disease 1	XL	*CLCN5*	ClC-5	Endosomal chloride channel	#300009
Dent disease 2	XL	*ORCL*	OCRL	Inositol polyphosphate 5-phosphatase	#300555
Lowe syndrome	XL	*OCRL*	OCRL	Inositol polyphosphate 5-phosphatase	#309000
Cystinuria	AD, AR	*SLC3A1*, *SLC7A9*	rBAT, b^(0,+)^AT1	Heavy and light subunits of the cystine and dibasic amino acids transporter	#220100
Proximal renal tubular acidosis–ocular anomaly syndrome	AR	*SLC4A4*	NBCe1	Sodium bicarbonate cotransporter	#604278
Osteopetrosis with renal tubular acidosis	AR	*CA2*	CAII	Carbonic Anhydrase 2	#259730
Fanconi renotubular syndrome 1	AD	*GATM*	GATM	Glycine Amidinotransferase	#134600
Fanconi renotubular syndrome 2	AR	*SLC34A1*	NaPi-2a	Sodium-dependent inorganic phosphate transporter	#613388
Fanconi renotubular syndrome 3	AD	*EHHADH*	PBFE	Peroxisomal enzyme of the β-oxidation pathway	#615605
Fanconi renotubular syndrome 4 with MODY	AD	*HNF4A*	HNF-4	Hepatocite nuclear factor	#616026
Imerslund-Grasbeck syndrome 1	AR	*CUBN*	Cubilin	Component of the receptor-mediated endocytosis complex	#261100
Imerslund-Grasbeck syndrome 2	AR	*AMN*	Amnionless	Component of the receptor-mediated endocytosis complex	#618882
Hypophosphatemic rickets with hypercalciuria	AR	*SLC34A3*	NaPi-2c	Sodium-dependent inorganic phosphate transporter	#241530
Nephropathic cystinosis	AR	*CTNS*	Cystinosin	Lysosomal cystine transporter	#219800
Hypophosphatemic rickets, autosomal dominant	AD	*FGF23*	FGF-23	Tubular phosphate reabsorption inhibitor	#193100
Hypophosphatemic rickets, autosomal recessive 1	AR	*DMP1*	DMP-1	Extracellular matrix protein from bone homeostasis	#241520
Hypophosphatemic rickets, autosomal recessive 2	AR	*ENPP1*	PC-1	Nucleotide phosphatase from bone homeostasis	#613312
Hypophosphatemic rickets, X-linked dominant	XL	*PHEX*	PEX	Transmembrane endopeptidase that degrades FGF-23	#307800
Hederitary glucosuria	AD, AR	*SLC5A2*	SGLT2	Sodium glucose cotransporter	#233100
Fanconi Bickel syndrome	AR	*SLC2A2*	GLUT2	Glucose transporter	#227810
Hartnup disorder	AR	*SLC6A19*	b^(0)^AT1	Neutral amino acids transporter	#234500
Donnai-Barrow syndrome	AR	*LRP2*	Megalin	Component of the receptor-mediated endocytosis complex	#222448
Hereditary renal hypouricaemia	AR, AR/AD	*SLC22A12*, *SLC2A9*	URAT1, GLUT9	Urate transporter, urate and glucose transporter	#220150, #612076
THICK ASCENDING LIMB OF THE LOOP OF HENLE					
Bartter syndrome type I	AR	*SLC12A1*	NKCC2	Sodium-potassium-chloride cotransporter	#601678
Bartter syndrome type II	AR	*KCNJ1*	ROMK	Potassium channel	#241200
Bartter syndrome type III	AR	*CLCNKB*	ClC-Kb	Voltage-gated chloride channel	#607364
Bartter syndrome type IVa	AR	*BSND*	Barttin	Essential subunit for CLC chloride channels	#302522
Bartter syndrome type IVb	Digenic	*CLCNKA + CLCNKB*	ClC-Ka + ClC-Kb	Voltage-gated chloride channel	#313090
Bartter syndrome type V	XL	*MAGED2*	MAGED2	Regulator of NKCC2	#300971
Hypomagnesemia type 3, with hypercalciuria and nephrocalcinosis	AR	*CLDN16*	Claudin 16	Component of tight junctions	#248250
Hypomagnesemia type 5, with hypercalciuria and nephrocalcinosis with severe ocular involvement	AR	*CLDN19*	Claudin 19	Component of tight junctions	#248190
Autosomal dominant hypocalcemia/Bartter-like syndrome	AD	*CASR*	CaSR	Calcium sensing receptor	#601198
HELIX syndrome	AR	*CLDN10*	Claudin 10	Component of tight junctions	#617671
DISTAL CONVOLUTED TUBULE					
Gitelman syndrome	AR	*SLC12A3*	NCC	Sodium chloride cotransporter	#263800
Pseudohypoaldosteronism type 2	AD	*WNK4*, *WNK1*, *KLHL3*, *CUL3*	WNK4, WNK1, KLHL3, CUL3	Serine/threonine kinase from the WNK-SPAK/OSR1 kinase cascade, *idem*, structural protein mediating E3 ubiquitin ligase pathway, component of the E3 ubiquitin-protein ligase complex	#614491, #614492, #614495, #614496
EAST/SeSAME syndrome	AR	*KCNJ10*	Kir4.1	Potassium channel	#602208
Hypomagnesemia type 1, with secondary hypocalcemia	AR	*TRPM6*	TRPM6	Magnesium channel	#602014
Hypomagnesemia with seizures and mental retardation type 1	AD/AR	*CNNM2*	Cyclin M2	Divalent metal cation transporter	#616418
Hypomagnesemia with seizures and mental retardation type 2	AD	*ATP1A1*	ATP1A1	α1 subunit of Na^+^/K^+^-ATPase	#618314
Hypomagnesemia type 2/Autosomal dominant primary hypomagnesemia hypocalciuria	AD/AR	*FXYD2*	FXYD2	γ subunit of Na^+^/K^+^-ATPase	#154020
Autosomal dominant hypomagnesemia	AD	*KCNA1*	Kv1.1	Voltage-gated potassium channel	#160120
Hypomagnesemia type 4	AR	*EGF*	EGF	Magnesiotropic hormone	#611718
COLLECTING DUCT					
Liddle syndrome	AD	*SCNN1A*, *SCNN1B*, *SCNN1G*	αENaC, βENaC, γENaC	α, β and γ subunits of the amiloride- sensitive sodium channel	#618126, #177200, #618114
Pseudohypoaldosteronism type 1B	AR	*SCNN1A*, *SCNN1B*, *SCNN1G*	αENaC, βENaC, γENaC	α, β and γ subunits of the amiloride- sensitive sodium channel	#264350, #620125, #620126
Pseudohypoaldosteronism type 1A	AD	*NR3C2*	MR	Mineralocorticoid receptor	#177735
Nephrogenic diabetes insipidus	XL, AR/AD	*AVPR2*, *AQP2*	V2R, aquaporin-2	Vasopressin receptor, collecting duct water channel	#304800, #125800
Familial hyperaldostoronism type I	AD	*CYP11B1*, *CYP11B2*	11-β-hydroxylase, aldosterone hydroxylase	Enzymes from the aldosterone and cortisol synthesis	#103900
Familial hyperaldosteronism type II	AD	*CLCN2*	ClC-2	Voltage-gated chloride channel	#605635
Familial hyperaldosteronism type III	AD	*KCNJ5*	Kir3.4	Inward-rectifier potassium channel	#313677
Familial hyperaldosteronism type IV	AD	*CACNA1H*	Cav3.2	Voltage-gated calcium channel	#617027
Distal renal tubular acidosis	AR/AD, AR, AR, AR, AR	*SLC4A1*, *ATP6V1B1*, *ATP6V0A4*, *FOXI1*, *WDR72*	AE1, ATP6V1B1, ATP6V0A4, FOXI1, WDR72	Anion exchanger, V-type ATPase subunit B1, V-type ATPase subunit A4, transcription factor required for *SLC4A1*, cytoplasmatic protein involved in intracellular trafficking	#611590, #267300, #602722

List of rare inherited renal tubulopathies grouped by the affected nephron segment with their inheritance, identified causal gene(s), affected protein(s), main protein function, and OMIM entry number. AD = autosomal dominant; AR = autosomal recessive; XL = X-linked inheritance.

## Data Availability

The original contributions presented in this study are included in the article. Further inquiries can be directed to the corresponding authors.

## Abbreviations

The following abbreviations are used in this manuscript:

PT	Proximal tubule
TAL	Thick ascending limb
DCT	Distal convoluted tubule
CD	Collecting duct
AD	Autosomal dominant
AR	Autosomal recessive
XL	X-linked inheritance
CKD	Chronic kidney disease
LMW	Low molecular weight
ESKD	End stage kidney disease
DD1	Dent disease type 1
DD2	Dent disease type 2
